# Patterned eruption following fishnet stocking use

**DOI:** 10.1016/j.jdcr.2024.11.037

**Published:** 2025-01-20

**Authors:** Vixey Silva, Bret-Ashleigh Coleman, Heather Kopecky, David Dorton, Carlos Nousari

**Affiliations:** a Department of Dermatology, HCA Healthcare/USF Morsani College of Medicine: Largo Medical Center Program, Largo, Florida; b Edward Via College of Osteopathic Medicine, Auburn, Alabama; c Dermpath Diagnostics, Pompano Beach, Florida

**Keywords:** Henoch-Schonlein purpura, immunoglobulin A vasculitis, Koebner phenomenon, purpura

## Case presentation

**Fig 1:**
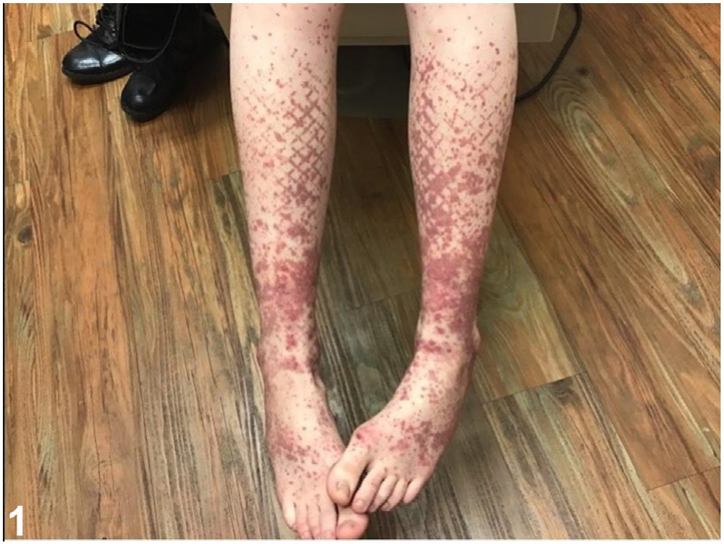

**Fig 2:**
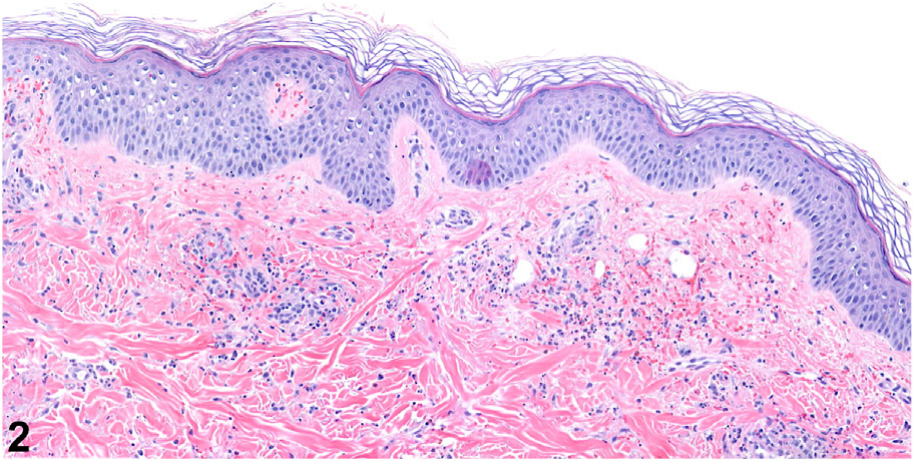

**Fig 3:**
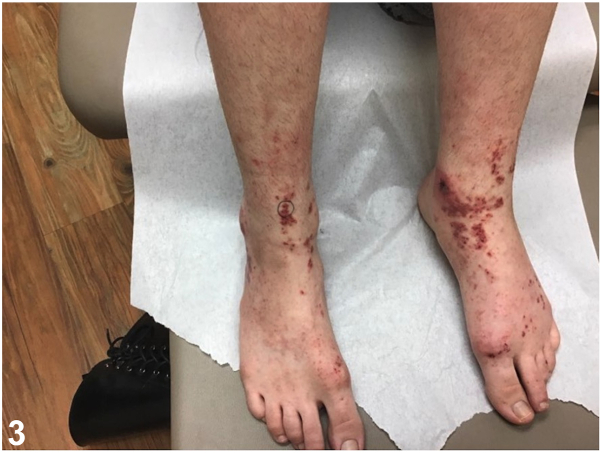

**Fig 4:**
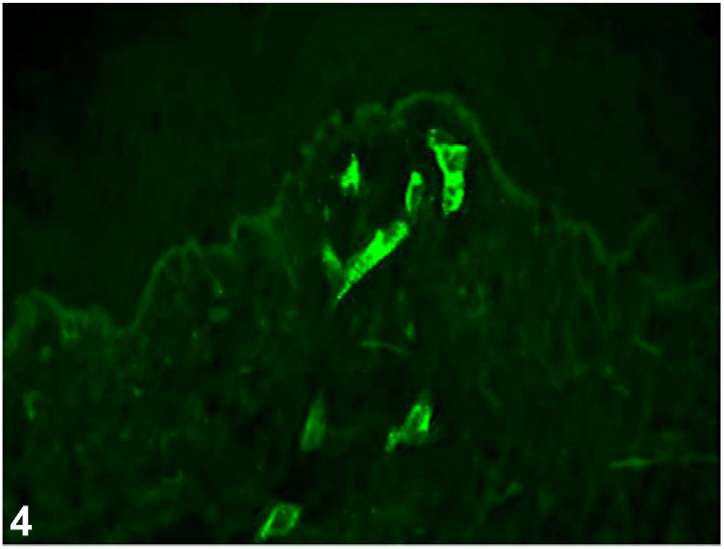

A female in her 20s presented with a 1-week history of violaceous purpuric, geometric, papules and plaques on bilateral shins, ankles, and feet (Fig 1). She reported associated pain, pruritus, malaise, and arthralgias. She denied recent illnesses and new medications. Of note, she wore fishnet stockings prior to eruption. Initial biopsy showed perivascular infiltrate with neutrophils, karryohexis, and extravasated red blood cells (Fig 2), antinuclear antibody was 1:80, complete metabolic profile and complete blood count were unremarkable. Betamethasone dipropionate 0.05% cream was prescribed. At 2-week follow-up, residual painful purpura persisted on ankles (Fig 3). A biopsy for direct immunofluorescence was performed and revealed granular IgA deposition around superficial papillary dermal blood vessels (Fig 4).

**Question 1: What is the most likely diagnosis?**

A. Lichen planus
B. IgA vasculitis
C. Urticarial vasculitis
D. Pigmented purpuric dermatosis
E. Dermatitis herpetiformis

**Answers:**

A. Lichen planus – Incorrect. Lichen planus presents with pruritic violaceous papules and plaques with Wickham striae on the lower extremities and exhibits the Koebner phenomenon. Direct immunofluorescence shows shaggy fibrinogen deposition along the basement membrane and Civatte bodies staining for IgM, IgG, IgA, or C3.^1^
B. IgA vasculitis – Correct. IgA vasculitis (IgAV), also known as Henoch-Schönlein purpura, presents with palpable purpura on the bilateral lower extremities and can rarely exhibit the Koebner phenomenon, as shown in this case.^2^^,^^3^ Malaise and arthralgias support the diagnosis, along with direct immunofluorescence findings of granular IgA within the walls of superficial papillary dermal blood vessels, as shown in Fig 3.^1^
C. Urticarial vasculitis – Incorrect. The pruritic papules and plaques in urticarial vasculitis typically affect the trunk and proximal extremities.^1^ Additionally, histology shows evidence of leukocytoclastic vasculitis with neutrophilic infiltrate and fibrinoid necrosis in vessels without IgA deposition.^1^
D. Pigmented purpuric dermatosis – Incorrect. Lesions often appear as purpuric papules, plaques, and petechiae with a characteristic “cayenne-pepper-like” appearance on the bilateral lower extremities, accompanied by pruritus and pain. Histopathology shows perivascular lymphohistiocytic infiltrate around blood vessels with endothelial cell swelling, without leukocytoclastic vasculitis. Fibrinogen can be seen on direct immunofluorescence; however, IgA deposition is not characteristically noted.^4^
E. Dermatitis herpetiformis – Incorrect. The eruption is pruritic but typically vesiculopapular, affecting extensor surfaces, buttocks, and scalp. Granular IgA deposition occurs within superficial dermal papillae, not within blood vessel walls.^1^

**Question 2: Which of the following conditions is least likely to present with palpable purpura?**

A. Cryoglobulinemia
B. Acute hemorrhagic edema of infancy
C. Microscopic polyangiitis
D. Thrombocytopenic purpura
E. Polyarteritis nodosa

**Answers:**

A. Cryoglobulinemia – Incorrect. Cryoglobulinemia is caused by cryoglobulins, immunoglobulins that precipitate at temperatures below 37 °C, leading to small vessel occlusion or immune complex-mediated vasculitis.^1^ It presents with palpable purpura on the lower extremities.
B. Acute hemorrhagic edema of infancy – Incorrect. This condition affects children and is characterized by leukocytoclasia of capillaries and venules.^1^ This results in palpable purpura and urticarial plaques that can be annular, polycyclic, and targetoid/cockade, often on the cheeks, ears, and extremities.
C. Microscopic polyangiitis – Incorrect. This is a perinuclear antineutrophil cytoplasmic antibody driven vasculitis that affects capillaries, venules, and medium-sized arteries.^1^ Clinically, patients present with palpable purpura, in addition to erythematous macules and patches, splinter hemorrhages, and ulcers.
D. Thrombocytopenic purpura – Correct. Thrombocytopenic purpura typically presents with nonpalpable purpura or petechiae due to thrombocytopenia and microangiopathic hemolytic anemia, rather than the palpable purpura seen in vasculitic conditions.
E. Polyarteritis nodosa – Incorrect. Polyarteritis nodosa is a vasculitis that affects medium-sized arteries and can present with palpable purpura, livedo racemosa, retiform purpura, and ulcers.^1^

**Question 3: In the absence of renal involvement, what is the most appropriate initial treatment for a patient with mild to moderate IgAV?**

A. High-dose systemic corticosteroids
B. Cyclosporine
C. Supportive care
D. Mycophenolate mofetil
E. Dapsone

**Answers:**

A. High-dose systemic corticosteroids – Incorrect. While corticosteroids may alleviate severe symptoms like arthralgias and abdominal pain, they are not the first choice for mild to moderate cases without renal involvement, as studies show no added benefit in preventing renal disease compared to supportive care.^5^ Even in cases with renal involvement, other treatments are often preferred due to corticosteroids' mixed efficacy and potential side effects.
B. Cyclosporine – Incorrect. Cyclosporine, a calcineurin inhibitor, is primarily used in cases with severe renal involvement.^5^ It is not indicated as an initial treatment for mild to moderate IgAV without renal symptoms.
C. Supportive care – Correct. In the absence of renal involvement, the most appropriate initial treatment for mild to moderate IgAV is supportive care.^5^ This approach is based on the typically self-limiting nature of the condition when renal function is not affected. Supportive care may include adequate hydration, rest, symptomatic pain control with nonsteroidal antiinflammatory drugs, monitoring for potential complications.^5^ More aggressive treatments are usually reserved for cases with severe symptoms or renal involvement.
D. Mycophenolate mofetil – Incorrect. This immunosuppressant is an effective treatment alternative for patients with renal involvement, particularly in cases of steroid-resistant nephrotic range proteinuria, and it has shown high efficacy compared to cyclophosphamide.^5^ However, it is not necessary or recommended for mild to moderate IgAV without renal symptoms.
E. Dapsone – Incorrect. While dapsone has shown efficacy in treating chronic recurrent skin eruptions associated with IgAV in some case studies, it is not the first-line treatment for acute onset of mild to moderate IgAV without renal involvement.^5^

## Conflicts of interest

None disclosed.
